# Influence of Preferments on the Physicochemical and Sensory Quality of Traditional Panettone

**DOI:** 10.3390/foods11172566

**Published:** 2022-08-25

**Authors:** Nicodemo C. Jamanca-Gonzales, Robert W. Ocrospoma-Dueñas, Norma B. Quintana-Salazar, Raúl Siche, Reynaldo J. Silva-Paz

**Affiliations:** 1 Departamento de Ingeniería, Escuela de Ingeniería en Industrias Alimentarias, Universidad Nacional de Barranca, Av. Toribio de Luzuriaga N° 376 Mz J- Urb. La Florida, Barranca 15169, Peru; 2 Facultad de Ciencias Agropecuarias, Universidad Nacional de Trujillo, Ciudad Universitaria, Trujillo 13011, Peru; 3 Facultad de Ingeniería y Arquitectura, Escuela de Ingeniería en Industrias Alimentarias, Universidad Peruana Unión, Av. Villa Unión S/N, Ñaña, Lima 15461, Peru

**Keywords:** panettone, preferment, biga, sponge, sourdough, texture, color

## Abstract

In Peru, panettones are consumed in July and December. The main ingredient of panettones is wheat flour, which can be replaced with substitute flours to improve their nutritional, textural and sensory properties. This study aimed to evaluate the physicochemical, textural and sensory characteristics of panettones produced with three preferments, namely, biga (PB), sourdough (PMM) and sponge (PE), with the substitution of red quinoa flour and amaranth compared with a commercial product (PC). A completely randomized design with four experimental treatments was used to evaluate the total carbohydrate content, ash, total energy, fat, moisture, protein, color and texture profile. In addition, sensory characteristics were evaluated by 80 consumers using the CATA method; the purchase intention and preference ranking were also investigated. The results showed better sensory characteristics of panettones produced with preferments compared with a commercial product with similar characteristics. The sponge preferment presented better sensory characteristics with a profile of sweet, spongy, vanilla odor and moist texture, along with greater acceptability, preference and purchase intention, followed closely by the biga. It was concluded that the sponge preferment presented better sensory properties, which were correlated with its texture profile as manifested by an intermediate hardness, good elasticity and cohesiveness, which translated into greater acceptability, preference and purchase intention.

## 1. Introduction

Panettone is a consumer product for the general public. It is produced in the form of a dome with wheat flour, eggs, fats, milk, essences, candied fruits, raisins, etc. It is traditionally consumed at Christmas time (late December), although in recent years, it has been consumed on national holidays (late July) such that the per capita consumption is 1.1 kg, placing Peru among one of the countries with the highest consumption in the world, surpassing Italy and Brazil [1]. In Italy, panettone is produced through artisanal or industrial processes, representing a very important economic element [2]. For the production of panettone, the main input is wheat flour; however, in recent years, studies have been conducted on the substitution by other flours, such as golden flaxseed flour (*Linum usitattissimum* L.), replacing up to 30% [3]. The incorporation of tuber purees, such as sweet potato and fruit pulps, influences the color profile [4] and mesquite flour (*Prosopis* spp.) decreases the resilience parameter and increases the adhesiveness, influencing the size of the alveoli, and therefore, the texture; however, it allows for an increase in nutritional and functional components, contributing to the enrichment of food [5]. The quality of panettone is defined by its physical and chemical properties (moisture, color, specific volume, density and texture), which influence consumer acceptability [6]. To improve the fermentation process, enzymes (amylase, xylanase and lipase) are used to influence the quality of the product [7]. The fermentation process is important because it influences the sensory attributes of the product, as well as the recovery of the yeast and lactic acid bacteria that are involved in sourdough fermentation and produce the desired aromas and/or aromatic precursors [8]. The use of selected microorganisms as a sourdough starter is a promising alternative for the production of panettone with good technological quality, microbiological stability, sensory differentiation and good consumer acceptance. Moreover, it is attractive, as it has no added preservatives [9].

In the production of panettone, it is important to consider the consistency and texture of the dough that is achieved during the fermentation process, which can involve one of the following options: (a) Sponge involves intermediate fermentation with dough that is made with flour, water and a little yeast and is made to ferment beforehand, while the rest of the ingredients are added later to make the final dough; it is also known as sourdough with yeast. (b) Biga consists of a firmer dough that is made as a starter dough; it is a drier preferment that is left to ferment at room temperature and is frequently used in bakeries. (c) Sourdough, known as the final fermentation, is a dough that contains a symbiotic culture of yeasts that are naturally present in foods, such as cereals (especially yeasts, such as *Saccharomyces cerevisiae*), and are responsible for the fermentation of wine and beer; sourdough also contains bacteria present in the environment [4]. The use of sourdough produced with *Lactobacillus fermentum* (LF) and *Wickerhamomyces anomallus* (WA) showed significant benefits in panettones by allowing for maintaining softness during storage and microbiological stability [9]; likewise, lactic acid bacteria produce a series of metabolites, such as organic acids, exopolysaccharides (EPS) and/or enzymes, that positively influence the texture and hardening of bread [10]. These fermentations influence the crumbs, and thus, the porosity, sponginess and specific volume of the product.

There is currently a trend to develop and innovate food products, seeking a balance between their nutritional and sensory characteristics. The nutritional content of foods is highly valued, and this information on labels is regulated by competent health authorities. The use of Andean cereals in the formulation of panettones increases the nutritional value, mainly of vegetable proteins, and is an alternative to animal proteins since they have good functional properties and high digestibility [11] to that extent there is the possibility of achieving enrichment through the use of various well-known Andean cereals, such as quinoa, kiwicha and cañihua. The acceptability of panettone depends directly on the sensory attributes perceived by the panelists; therefore, the sensory evaluation focused on finding a connection with consumer behavior [12] and obtaining their sensory profile. In recent years, the check all that apply (CATA) test has been increasingly used for consumer-based sensory characterization; it represents a rapid approach to collecting information about the sensory characteristics of products directly from consumers [13,14]. This method consists of reviewing the attributes they consider appropriate to describe the product in a CATA multiple-choice questionnaire. The responses were shown to be able to discriminate between products and to be reproducible [13,14]. The sensory aspect of the product is fundamental; thus, the CATA test allows for distinguishing the aroma and taste of different panettones [9]. Currently, it is necessary to implement rapid descriptive tests with consumers that require less training; to this extent, the CATA test is better for building a panorama of products and highlighting groups of products with large sensory differences compared with other tests, such as napping, flash profile or RATA (rate all that apply) [15].

The objective of this study was to evaluate the physicochemical, textural and sensory characteristics of panettones produced with three preferments, namely, biga, sourdough and sponge, with the substitution of red quinoa flour and kiwicha compared with a commercial product.

## 2. Materials and Methods

### 2.1. Panettone Raw Material

The panettone was made in a bakery laboratory of the EP Ingeniería de Industrias Alimentarias. For the preparation of the panettone, the ingredients were incorporated according to the pre-established formulation in the following proportions: Partners wheat panettone flour (30.00%), Fleishmann fresh yeast made up of living microorganisms of the genus Saccharomyces cerevisiae according to the type of preferment, Paramonga refined white sugar (9.60%), Puratos wheat gluten (2.40%), drinking water (15.00%), Mojapan Plus industrial margarine (3.60%), Fruit Dor dehydrated cranberry (10.49%), La Reina de Chulucanas honey (1.80%), Fratello glucose (0.60%), S500 Acti-plus-SB Puratos improver (0.30%), La Campiña powdered milk (1.20%), Super Suave Fresh Industrial improver (0.30%), Emsal sea salt (0.30%), Aromatic anti-mold (0.09%), La Calera egg yolks (3.60%), Mixo paste emulsifier (0.60%), Frutaron vanilla essence (0.09%), Puratos panettone essence (0.27%), Cuzqueña black beer (1.50%), bulk red quinoa (1.50%), bulk kiwicha (1.50%), fruit candied Tomasino (7.50%) and bulk pecans (3.00%). This formulation was kept constant between three treatments. Red quinoa and kiwicha were purchased in the model market of Barranca–Peru from the Peruvian highlands and where were incorporated as broken grains after selection, classification and precooking at 100 °C for 2 min. The commercial product consisted of a La Casa de la Salud wholemeal panettone, whose formulation used the following ingredients: wholemeal flour, eggs, vegetable shortening, soy lecithin, raisins, chestnuts, pecans and cane syrup.

### 2.2. Panettone Dough Preference

The creation of preferments is an important procedure in the creation of panettones, which directly influences the texture and sensory attributes of the product. We worked with three production preferments: preferment biga (PB), which was prepared with 30% total flour, 50% water and 18% fresh yeast; preferment masa madre (PMM), which was processed with 30% total flour, 9% fresh yeast and 9% sourdough produced with own yeasts (natural) of wheat flour with an age of 5 years and provided by a specialist master baker; and preferment type sponge (PE), which was processed with 60% total wheat flour in the base formulation, 60% drinking water and 9% fresh yeast. There was also a control sample (PC), which was a commercial product of the whole wheat panettone type that was made with whole wheat flour, eggs, vegetable shortening, soy lecithin, raisins, chestnuts, pecans and cane honey; a total of four samples were obtained.

### 2.3. Processing of Panettone

The panettone was made by mixing the preferment, adding 40% (in dough) wheat flour, dry yeast, sugar, gluten, margarine and drinking water. The preferment was mixed with a 25 kg Nova industrial mixer at a speed of 18 rpm for 10 min until a smooth and elastic dough consistency was obtained. The mixture was rested for 40 min and the rest of the ingredients were added, mixing for 20 min until the dough had a smooth consistency. Then, the dough was rested for 20 min before dividing it into 900 g portions. Then, it was rounded and placed in cylindrical pyrotes. The products were placed in the fermentation chamber for 1 h at 28 °C and 83% RH, then baked at 140 °C for 50 min. The product was cooled to room temperature (18–25 °C approximately), suspended vertically on hooks, bagged, sanitized with antimould spray, closed, boxed and stored in a cool environment.

### 2.4. Physicochemical Analysis and Imaging of the Crumb Structure

The ash [16], fat [17], moisture [18], protein [19] and total carbohydrates were calculated by difference [20], while the total energy was calculated using the conversion factors recommended by the FAO [21]. The physicochemical analyzes applied to the panettone samples were carried out in duplicates. To obtain images, cross-sections were made in the sample with an area of 200 × 250 mm from the central region at a distance of 20 cm and a height of 10 cm from the base using a Nikon D7500 camera with 8/d illumination and a D50 light source.

### 2.5. Specific Volume and Density

The samples were weighed on a Sartorius brand analytical balance (g) and the bread volume (mL) was determined using the canola displacement method [6]. Measurements of specific volume and density were carried out in quadruplicate. The specific volume (mL/g) = (volume (mL))/(weight (g)) and the density (g/mL) = (weight (g))/(volume (mL)).

### 2.6. Texture Test

Texture profile analysis (TPA) parameters were determined using a Stable Micro Systems Texture Analyser TA HD Plus. The test conditions were the following: probe compression plates P/45 Ø 4.5 cm, double compression, speed 2.0 mm/s, deformation 40%, load cell 5 kg, evaluating hardness (maximum deformation force during the first mastication cycle), elasticity (relative to the speed of recovery of the deformation after the application of a force and the degree of said recovery), cohesiveness (the ratio of the positive force during the second compression to that during the first compression), chewiness (calculated parameter: gumminess × springiness (equivalent to hardness × cohesiveness × springiness)) and resilience (the greater the resilience, the more capacity the food has to return to its original form) [6]. Data recording and analysis of the texture profile were performed by using Exponent version 4 software [22]. The samples were cut into slices of 45 mm in diameter and 45 mm in height (cylindrical shape) and extracted from the central part (crumb); the measurements were performed as quickly as possible to avoid moisture loss. Texture parameters were measured in quadruplicate replicates.

### 2.7. Crumb and Crust Color Test

The color parameters of the crumb and rind were determined with a Colorimeter model 3NH with an observation angle of 10° and an Illuminant D65. The color was recorded using CIE-L* a* b* with a uniform color space (CIE-Lab), where L* indicates the lightness, a* indicates the hue on a green (–) to red (+) axis and b* indicates the hue on a blue (–) to yellow (+) axis [6,20], as well as CIE-L*C*h*, where L* denotes the lightness, C* denotes the chroma and h* denotes the hue or angle of a polar measurement. The chroma value C* = (a^*2^ + b^*2^)^0.5^ and the hue angle h* = arctangent(b*/a*). The color measurement of the samples was performed in triplicate [23].

### 2.8. Sensory Analysis

A consumer study was conducted with 80 consumers (38.75% men and 61.25% women) between 19 and 61 years old. All the participants consumed panettone on a regular basis. The tests were carried out in the first fifteen days of December 2021. The samples were manually cut into triangular pieces of 50 ± 6 g m and presented in a polypropylene plate (P15) of 10 cm in diameter. The samples were coded with three random numbers. The evaluation was carried out at room temperature in individual cabins. Each consumer was given the evaluation sheet where the attributes were randomized and they were asked to indicate the sensory characteristics of the panettone using the CATA method. Eighteen sensory terms related to smell, color, taste, texture and general appearance (alcohol smell, vanilla smell, fruit smell, fermented smell, nutty smell, shiny, pale, sweet, bitter, sour, strange, spongy, moist, greasy, soft, fibrous, dry/hard, sticky or melcochado) were used, which were previously identified by bakery experts. In addition, they were asked to indicate acceptability using a hedonic scale, their purchase intention with a five-point scale and, finally, they carried out a preference ranking test. The samples were presented in a monadic form and table water was used between each sample to clean the palate.

### 2.9. Statistical Analysis

A completely randomized design was applied, with the types of panettone as the factor and the physicochemical analysis, volume, density, color and texture as the response variables. For the sensory analysis, a completely randomized block design was used for acceptability and purchase intention, where the Friedman test was applied. In the CATA method, Cochran’s Q test and correspondence analysis were performed. The results are expressed as the mean value ± standard deviation (SD) or frequencies according to the type of data. In the case of finding significance, Tukey’s mean comparison was performed with 95% confidence. The data were processed using Xlstat software—trial version.

## 3. Results and Discussion

### 3.1. Chemical Composition

As shown in Table 1, significant differences (*p* ≤ 0.05) were observed in the centesimal composition, with the exception of density and specific volume (*p* ≥ 0.05). Ash, fat, protein and energy contents were higher for the PC sample and lower for moisture and total carbohydrates. The PB and PMM samples recorded the highest carbohydrate and moisture contents, respectively. The chemical compositions of the different panettones were specifically due to the ingredients used, including the type of flour used in the process (paneton flour), which is characterized by a higher gluten content. The most important results were related to the ash, moisture and protein contents. The ash content in panettone was related to the quality of the flour used as raw material and was below the maximum of 3%, as established by the Peruvian legislation for this type of product [24]. Moisture is an important property in panettones because it is associated with softness and ease of chewing; it can vary according to the type of product and process conditions [6]. The samples presented moisture values between 23.51 and 27.70%, which are within the standards allowed by Peruvian legislation (maximum 40%) [24], and the protein values were between 8.90 and 10.40%, which are values that were close to those reached by Bigne et al. [5] in panettone enriched with mesquite (*Prosopis alba*). In addition, they indicated that lower humidity increases the total solids, mainly protein content. Moisture allows for maintaining the characteristics of panettone under storage, packaging, temperature and relative humidity conditions [25]. The energy value was related to the sum of the macronutrients composed of carbohydrates, proteins and fats, with PC having the highest energy value due to the ingredients used in its preparation, such as wholemeal flour, eggs, vegetable shortening, soya lecithin, raisins, chestnuts, pecans and cane honey, as evidenced by its low moisture content.

**Figure 1:**
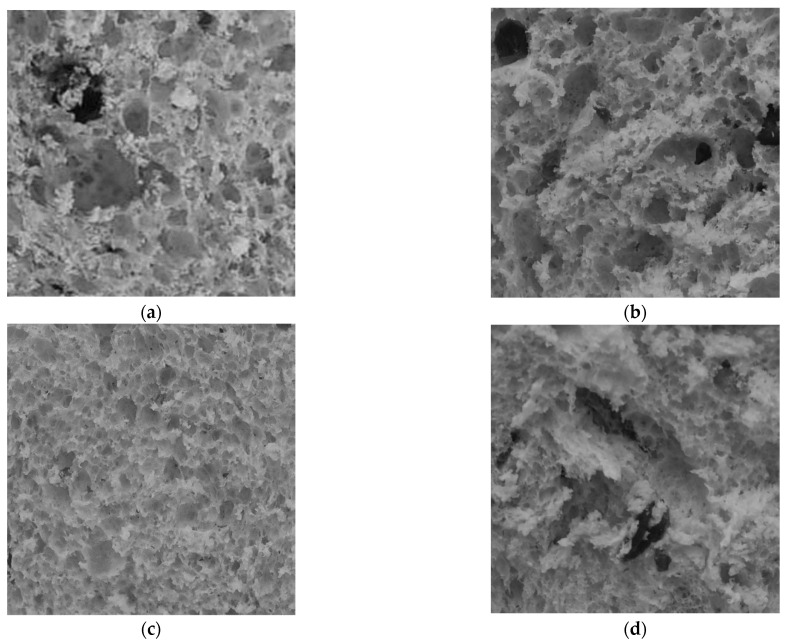
Images of the porous structure of the crumb of the panettone samples (area 60 × 60 mm): (**a**)PC, (**b**) PB, (**c**) PMM and (**d**) PE. The image is taken at a 10× zoom expressed on a scale of 1 mm.

Regarding the structural aspect, panettone consists of a porous structure (alveoli) and a solid portion, whose sum will influence the total volume of the product since both the specific volume and the density indicate the relationship between the solids and the air fraction [26]. According to the results, no significant values were observed for the specific volume and density due to the similarity in the formulations and elaboration of the three types of preferment (PB, PMM and PE) and the commercial sample (PC). In general, the samples were characterized by presenting a highly aerated structure; in addition, the presence of candied fruits, raisins and dried cranberries influenced the actual volume of the crumb of the panettone [6]. Quality panettone usually has a structured crumb with high porosity and regular cells (Figure 1). However, each type of product has its own configuration of cells or alveoli, which are the holes in the crumb, which originate when air is trapped between the gluten networks, and therefore, there is no single standard applicable to all products [25] whose level and thickness characterize bakery products that undergo volumetric expansion during baking [26,27]. All the panettone samples showed a very porous crumb structure (Figure 1). The results showed that there was product variability, even in the commercial sample, and it was difficult to obtain baked products with homogeneous porosity characteristics. The causes of this variability were related to the mechanical characteristics of gluten, which are linked to its ability to stretch, breakage during baking, starch softening during baking and too many starch granules [28]. Each type of product has its own special cell structure, and therefore, there is no single pattern that can be generalized to all products [29]. Porosity is higher with increasing heating rate and steam baking; furthermore, non-uniformity in local expansion during baking is a result of density variability in different areas of the product [30], which is influenced by solid components such as candied fruits, raisins and dried cranberries.

### 3.2. Instrumental Texture Analysis

Table 2 shows that significant differences were found (*p* ≤ 0.05) regarding the elasticity and resilience in the texture profile, while there were no significant differences (*p* ≥ 0.05) in hardness, cohesiveness and chewiness. The PB sample presented lower values in elasticity and resilience compared with the other samples; however, the PC, PMM and PE samples did not present significant differences between them. Mechanical parameters are important in bakery products. The texture of the crumb is related to the mechanical properties and the maximum value of the force measured for bakery products depends directly on the formulation [5,6]. Some ingredients, such as flour, sugars, fats, emulsifiers, enzymes, gluten and flour improvers, together with the humidity of the dough and storage (product manufacturing time and packaging), affect the quality of the final product [31]. Regarding the texture profile, hardness, cohesiveness and chewiness did not show significant differences, although a slight tendency to increase these parameters was observed in PC, and lower values were found for PMM and PB in relation to the hardness and cohesiveness/chewiness, respectively. These values were in the ranges reported by Valcarcel-Yamani [6] regarding hardness, elasticity, cohesiveness, chewiness and resilience, although lower than those of Bigne et al. [5]. The hardness or softness of the crumb is a property that receives more attention in the evaluation of bakery products due to its close association with the human perception of freshness [25]. The results of the elasticity tests indicated higher values for PC, PMM and PE, and lower values for PB. Low values of elasticity and high chewiness are usually indicators of storage time [31]. Lower moisture is related to aged and dry bakery products that require more salivation and chewing, although chewiness is directly related to the presence of fat and sugar [7].

### 3.3. Colorimetric Parameters

Table 3 shows that the colorimetric parameters showed significant differences (*p* ≤ 0.05) in all parameters, namely, L*, a*, b*, C* and h*, both in the rind and crumb. The rind of the PC and PE samples showed lower values in all colorimetric parameters and an inverse behavior was observed for PB and PMM. Regarding the crumb, the PC sample presented higher values in the parameters a* and C*, while being lower in h*. In addition, the brightness and chromaticity were statistically similar for PC, PB and PMM. The values of the color parameters of the crust and crumb of the panettone (Table 3) were very varied, with the crumb presenting a higher brightness than the crust, which was linked to chemical changes, such as the Maillard reaction that occurs during the baking process due to the presence of free amino acids that react with reducing sugars [32]. The rind color parameters, namely, L* (which indicates a tendency to lightness between black and white), a* and b*, were very similar to those determined by Valcarcel-Yamani et al. [6]. These variations occur during the cooking process when increasing the temperature, which leads to the inactivation of yeast and enzymes, starch gelatinization, changes in proteins (mainly gluten) and the release of water from the surface, causing crust development and the Maillard reaction responsible for color and flavor. In this process, the dark color of the crust intensifies, which is influenced by high oven temperatures or overcooking. However, a light color is a sign of long fermentation doughs, a cold oven, or even insufficient baking time [31,33]. As for the crumb color parameters, they presented values that were very close to those reported by Valcarcel-Yamani et al. [6]. These values indicate higher light reflectance in lighter-colored products with lower sugar content or heat-activated ingredient interactions, where these values correspond to typical bakery products rich in proteins, sugars and carotenoids. The values of b* for panettones are indicators of lower intensities of a yellow tone, which is explained by having avoided the use of synthetic dyes that are usually used in commercial products. According to Esteller et al. [31], some variations in a* and b* values may also be related to porosity, and in the case of panettone, the presence of candied fruit and raisins may have a direct influence.

### 3.4. Sensory Analysis

Table 4 shows that out of the 18 attributes studied, 10 descriptors (alcohol odor, fruity odor, nutty aroma, bright, pale, sweet, bitter, strange flavor, mild and dry/hard) showed significant differences (*p* ≤ 0.05). Therefore, the use of the CATA method allowed the consumers to find sensory attributes to describe and differentiate the evaluated samples. The applied sensory survey and dynamic table of sensory attributes selected by con-sumers can be found in Supplementary material (Files S1 and S2).

Different sensory profiles were described for different panettones (Table 4). In the PC sample, consumers indicated the attributes of an alcoholic odor and a strange taste more frequently compared with the PB, PMM and PE samples, which did not show differences between them. The PMM and PE samples were characterized by fruity and pale odors compared with PC and PB. In soft and sweet attributes, the PB, PMM and PE samples did not show significant differences between them, although they differed from PC (*p* ≤ 0.05). Samples PC, PB and PMM were similar regarding the terms shiny and nutty aroma. The term bitter was used with a higher frequency for PC and PB than for the other samples. The highest frequency of the attribute dry/hard was for PC and PMM.

**Figure 2:**
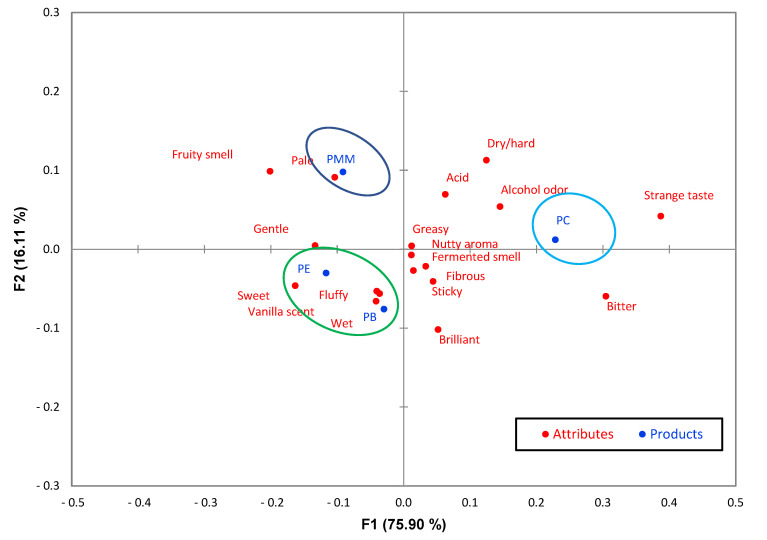
Correspondence analysis graph using the CATA method.

Figure 2 shows the correspondence analysis of the samples and the attributes that explain in two dimensions 92% of the total variability of the data. The formation of three groups was observed: the first group was formed by the PC sample, the second group by PMM and the third group by PE and PB. The PC sample was perceived as having a strange taste, alcoholic odor, acidic and dry/hard. PMM was described as pale and having a fruity odor. The PE and PB samples were characterized as spongy, sweet, moist and having a vanilla odor, with the latter group having the best sensory profile. On the other hand, the limited number of attributes and the absence of descriptive analysis in this type of product to define sensory characteristics and obtain accurate, robust and reliable quantifications made it difficult to compare the results with other research. However, the CATA method allowed the consumers to find a profile of panettone quickly, using less time and reducing the expenses compared with using descriptive analysis [14,34,35]. The application of the quantitative descriptive method would allow for obtaining more research results based on evaluating panettones with a trained panel.

**Figure 3:**
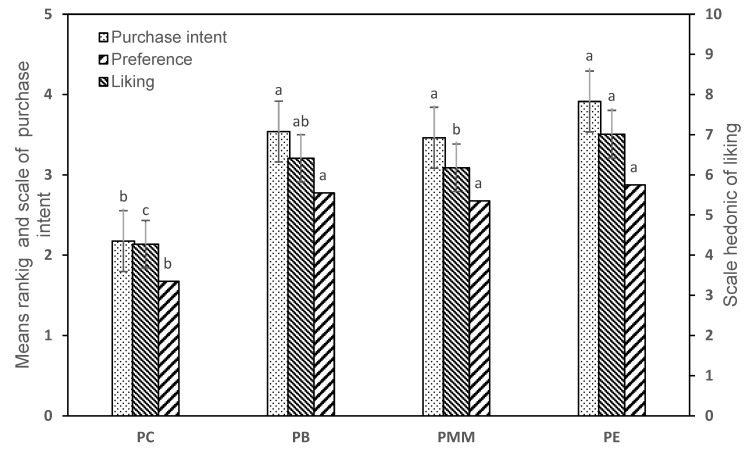
Acceptability, purchase intention and sample preference. ^a,b,c^ Different letters indicate significant differences (p ≤ 0.05) according to the Tukey test (acceptability and purchase intention) and Friedman test (preference).

Figure 3 presents the results of acceptability, purchase intention and preference, where it can be seen that there was a significant difference (*p* < 0.05). In terms of acceptability, the PMM, PE and PB samples were similar to each other, which were qualified as “I like it”, unlike the PC sample, which was described as “I don’t like it”. Similar behavior was observed for purchase intention, where consumers were possibly willing to buy the PE, PB and PMM samples (*p* > 0.05) and probably would not buy the PC sample. Regarding preference, the PB sample showed a higher preference, although it was not significantly different from PMM and PE. Therefore, the type of preferment did not directly influence the consumer preference.

## 4. Conclusions

The use of preferments influenced the textural and sensory characteristics of the panettones, reducing hardness (greater softness), improving its chewiness and resistance, and achieving a more porous structure. The physicochemical characteristics depended on the formulation and production process, where significant differences were found in all the parameters studied, except for density and specific volume. The sensory profile of the samples formed three groups: the first group was made up of the PE and PB samples, which were characterized by being spongy, sweet, moist and smelling of vanilla; the second group was constituted by PC, which was described with a strange taste, an alcoholic smell, acidic and dry; and the third group formed by PMM was characterized by a pale color and a fruity smell. The panettones presented significant differences in acceptability, purchase intention and sample preference. In relation to acceptability, the PE and PB samples showed the highest acceptability (“I like it a lot”), while PC had the lowest acceptability (“I dislike it a little”). The purchase intention results had the same behavior, where the PE sample was the one with the greatest purchase intention. In relation to preference, the PE sample was preferred by consumers, although its values were statistically similar to PB and PMM, while the PC sample presented the lowest preference. In general, the results indicated an improvement in the physicochemical and sensory parameters compared with the commercial sample; therefore, the use of preferments is viable for the development of panettones. It is recommended to study the nutritional value with a focus on protein quality and its sensory perception by consumers by applying different modern sensory methods.

## Figures and Tables

**Table 1:** Proximal chemical composition of 100 g of panettone.

Sample	Ash (g)	Fat (g)	Moisture (g)	Protein (g)	Total Carbohydrates (g)	Energy (kcal)	Density (g/mL)	Specific Volume (mL/g)
PC	1.84 ± 0.014 ^a^	12.26 ± 0.001 ^a^	23.51 ± 0.028 ^d^	10.40 ± 0.0141 ^a^	51.97 ± 0.092 ^d^	359.90 ± 0.170 ^a^	0.310 ± 0.027 ^a^	3.243 ± 0.303 ^a^
PB	1.08 ± 0.014 ^b^	6.52 ± 0.007 ^c^	26.76 ± 0.028 ^b^	9.44 ± 0.0424 ^b^	56.20 ± 0.010 ^a^	321.24 ± 0.170 ^c^	0.372 ± 0.137 ^a^	3.050 ± 1.332 ^a^
PMM	1.10 ± 0.028 ^b^	6.58 ± 0.021 ^c^	27.70 ± 0.014 ^a^	9.35 ± 0.0212 ^b^	55.27 ± 0.084 ^c^	317.68 ± 0.064 ^d^	0.411 ± 0.080 ^a^	2.521 ± 0.577 ^a^
PE	1.07 ± 0.028 ^b^	7.76 ± 0.021 ^b^	25.63 ± 0.028 ^c^	8.90 ± 0.0283 ^c^	56.65 ± 0.049 ^b^	331.98 ± 0.106 ^b^	0.382 ± 0.090 ^a^	2.767 ± 0.792 ^a^

Values followed by different letters in the same column showed significant differences (*p* ≤ 0.05) according to Tukey’s test.

**Table 2:** Instrumental texture profile (TPA) of panettone.

Sample	Hardness (N)	Elasticity	Cohesiveness	Chewiness (N)	Resilience
PC	5.09 ± 0.737 ^a^	1.20 ± 0.307 ^ab^	0.56 ± 0.025 ^a^	3.34 ± 0.716 ^a^	0.17 ± 0.008 ^ab^
PB	5.06 ± 1.317 ^a^	0.91 ± 0.053 ^b^	0.49 ± 0.038 ^a^	2.25 ± 0.349 ^a^	0.14 ± 0.013 ^b^
PMM	3.90 ± 0.880 ^a^	1.35 ± 0.268 ^a^	0.55 ± 0.057 ^a^	2.90 ± 1.034 ^a^	0.17 ± 0.024 ^a^
PE	4.73 ± 0.560 ^a^	0.98 ± 0.009 ^ab^	0.54 ± 0.035 ^a^	2.52 ± 0.319 ^a^	0.15 ± 0.009 ^ab^

Values followed by different letters in the same column showed significant differences (*p* ≤ 0.05) according to Tukey’s test.

**Table 3:** Color of the crumb and crust of panettone.

Sample	Crust				
	L*	a*	b*	C*	h*
PC	36.78 ± 1.452 ^b^	16.41 ± 0.920 ^b^	23.38 ± 0.911 ^b^	28.57 ± 1.247 ^b^	54.95 ± 0.675 ^b^
PB	44.82 ± 1.845 ^a^	19.49 ± 0.973 ^a^	34.15 ± 1.166 ^a^	39.33 ± 1.189 ^a^	60.29 ± 1.387 ^a^
PMM	44.74 ± 1.034 ^a^	18.51 ± 1.330 ^ab^	33.21 ± 0.845 ^a^	38.03 ± 1.156 ^a^	60.89 ± 1.613 ^a^
PE	36.20 ± 3.280 ^b^	18.13 ± 0.317 ^ab^	25.41 ± 2.800 ^b^	31.25 ± 2.190 ^b^	54.32 ± 3.220 ^b^
	**Crumb**				
PC	71.05 ± 2.360 ^ab^	10.48 ± 0.309 ^a^	33.94 ± 0.327 ^a^	35.52 ± 0.384 ^a^	72.85 ± 0.378 ^b^
PB	73.31 ± 2.820 ^a^	7.11 ± 0.967 ^b^	29.13 ± 1.480 ^ab^	29.99 ± 1.641 ^b^	76.34 ± 1.271 ^ab^
PMM	69.77 ± 2.010 ^ab^	6.34 ± 1.774 ^b^	27.56 ± 3.670 ^b^	28.30 ± 3.940 ^b^	77.25 ± 2.270 ^a^
PE	68.31 ± 1.381 ^b^	6.57 ± 1.159 ^b^	27.16 ± 0.966 ^b^	27.95 ± 1.168 ^b^	76.45 ± 1.959 ^ab^

Values followed by different letters in the same column showed significant differences (*p* ≤ 0.05) according to Tukey’s test.

**Table 4:** Sensory analysis results from Cochran’s Q test after using the CATA method.

Attributes	*p*-Value	PC	PB	PMM	PE
Alcohol odor	***0.002***	0.550 ^b^	0.425 ^ab^	0.338 ^a^	0.425 ^ab^
Vanilla scent	0.240	0.500 ^a^	0.575 ^a^	0.550 ^a^	0.475 ^a^
Fruity smell	***0.000***	0.388 ^a^	0.525 ^ab^	0.613 ^b^	0.688 ^b^
Fermented smell	0.781	0.438 ^a^	0.413 ^a^	0.425 ^a^	0.388 ^a^
Nutty aroma	***0.011***	0.663 ^ab^	0.563 ^ab^	0.700 ^b^	0.525 ^a^
Brilliant	***0.005***	0.538 ^ab^	0.613 ^b^	0.450 ^ab^	0.413 ^a^
Pale	***0.007***	0.600 ^a^	0.613 ^a^	0.738 ^a^	0.788 ^a^
Sweet	***0.000***	0.563 ^a^	0.825 ^b^	0.888 ^b^	0.750 ^b^
Bitter	***0.000***	0.450 ^b^	0.300 ^ab^	0.225 ^a^	0.200 ^a^
Acid	0.337	0.350 ^a^	0.300 ^a^	0.263 ^a^	0.325 ^a^
Strange taste	***0.000***	0.625 ^b^	0.300 ^a^	0.263 ^a^	0.275 ^a^
Fluffy	0.099	0.663 ^a^	0.763 ^a^	0.738 ^a^	0.638 ^a^
Wet	0.172	0.363 ^a^	0.438 ^a^	0.400 ^a^	0.350 ^a^
Greasy	0.677	0.325 ^a^	0.338 ^a^	0.288 ^a^	0.313 ^a^
Gentle	***0.008***	0.463 ^a^	0.625 ^a^	0.625 ^a^	0.625 ^a^
Fibrous	0.090	0.538 ^a^	0.463 ^a^	0.525 ^a^	0.425 ^a^
Dry/hard	***0.004***	0.613 ^b^	0.438 ^ab^	0.388 ^a^	0.525 ^ab^
Sticky	0.181	0.313 ^a^	0.325 ^a^	0.263 ^a^	0.263 ^a^

Values followed by different letters in the same column showed significant differences (*p* ≤ 0.05) according to Cochran’s test.

## Data Availability

The data is contained in the article.

## Supplementary Materials

The following are available online at https://www.mdpi.com/article/10.3390/foods11172566/s1, File S1: Sensory evaluation by cata method and purchase intention test; File S2: Sensory eval-uation preference ranking test.
