# Supplementary material for: Influence of Preferments on the Physicochemical and Sensory Quality of Traditional Panettone
Source: Foods. 2022 Aug 25;11(17):2566. doi: 10.3390/foods11172566 (PMC9455473; doi:10.3390/foods11172566)
Supplement: Supplementary file 1 [file foods-11-02566-s001.zip › foods-1838391-supplementary.pdf]

File S1 Sensory evaluation by cata method and purchase intention test

Age: ______________________________ Date: ______________________________
Product name: **Panettone** Sex: M ( ) F ( )

Instruccions:

- You will receive four panettone samples with random codes.
- Please try the samples in the order you want and answer the question using the list of words presented.
- Rinse your mouth with a little water between samples.

Sample code: ____________________

How much do you like this panettone?

☐ ☐ ☐ ☐ ☐ ☐ ☐ ☐ ☐

I really dislike — It does not matter to me — I really like

Would you buy this product?

☐ ☐ ☐ ☐ ☐

I wouldn't buy it — Maybe i would buy it — Yes, i would

Mark all the words that you consider appropriate to describe this panettone

SMELL		COLOR		TEXTURE	
**Alcohol smell**	☐	**Shiny**	☐	**Spongy**	☐
**Vanilla smell**	☐	**Pale**	☐	**Moist**	☐
**Fruit smell**	☐	**TASTE**		**Greasy**	☐
**Fermented smell**	☐	**Sweet**	☐	**Soft**	☐
**Nutty smell**	☐	**Bitter**	☐	**Fibrous**	☐
		**Sour**	☐	**Dry/hard**	☐
		**Strange**	☐	**Stick (melcochado)**	☐

Comments:

____

____

____

Thank you for your participation!

File S2 Sensory evaluation preference ranking test

Age: ______________________________ Date: ______________________________

Product name: **Panettone** Sex: M ( ) F ( )

<u>Instruccions:</u>

- You will receive four panettone samples with random codes
- Please locate the samples by writing the codes in order from least to most preferred:

------------------- −

------------------- +

Thank you for your participation!

Contingency table built from CATA data:

Products\Dimensions	Alcohol odor	Vanilla scent	Fruity smell	Fermented smell	Nutty aroma	Brilliant	Pale	Sweet	Bitter	Acid	Strange taste	Fluffy	Wet	Greasy	Gentle	Fibrous	Dry/hard	Sticky
**PB**	34	46	42	33	45	49	49	66	24	24	24	61	35	27	50	37	35	26
**PE**	27	44	49	34	56	36	59	71	18	21	21	59	32	23	50	42	31	21
**PMM**	34	38	55	31	42	33	63	60	16	26	22	51	28	25	50	34	42	21
**PC**	44	40	31	35	53	43	48	45	36	28	50	53	29	26	37	43	49	25
